# Detecting antibody reactivities in Phage ImmunoPrecipitation Sequencing data

**DOI:** 10.1186/s12864-022-08869-y

**Published:** 2022-09-15

**Authors:** Athena Chen, Kai Kammers, H Benjamin Larman, Robert B. Scharpf, Ingo Ruczinski

**Affiliations:** 1 Department of Biostatistics, Johns Hopkins Bloomberg School of Public Health, Baltimore, MD USA; 2 Department of Oncology, Johns Hopkins University School of Medicine, Baltimore, USA; 3 Department of Pathology and the Institute for Cell Engineering, Johns Hopkins University School of Medicine, Baltimore, USA

**Keywords:** Antobodies, Bayesian Model, Peptides, Phage ImmunoPrecipitation Sequencing, Reactivity

## Abstract

Phage ImmunoPrecipitation Sequencing (PhIP-Seq) is a recently developed technology to assess antibody reactivity, quantifying antibody binding towards hundreds of thousands of candidate epitopes. The output from PhIP-Seq experiments are read count matrices, similar to RNA-Seq data; however some important differences do exist. In this manuscript we investigated whether the publicly available method edgeR (Robinson et al., Bioinformatics 26(1):139–140, 2010) for normalization and analysis of RNA-Seq data is also suitable for PhIP-Seq data. We find that edgeR is remarkably effective, but improvements can be made and introduce a Bayesian framework specifically tailored for data from PhIP-Seq experiments (Bayesian Enrichment Estimation in R, BEER).

**Supplementary Information:**

The online version contains supplementary material available at 10.1186/s12864-022-08869-y.

## Introduction

Because of their high abundance, easy accessibility in peripheral blood, and relative stability ex vivo, antibodies serve as excellent records of environmental exposures and immune responses. While several multiplexed methods have been developed to assess antibody binding specificities, Phage Immuno-Precipitation Sequencing (PhIP-Seq) is the most efficient technique available for assessing antibody binding to hundreds of thousands of peptides at cohort scale [1–3]. PhIP-Seq uses oligonucleotide library synthesis to encode proteome spanning peptide libraries for display on bacteriophages. These libraries are immunocaptured using an individual’s serum antibodies, and the antibody-bound library members are identified by high throughput DNA sequencing. The VirScan [3] assay uses the PhIP-Seq method to quantify antibody binding to around 100,000 peptides spanning the genomes of more than 200 viruses that infect humans. Other commonly used libraries include the AllerScan [4] and ToxScan libraries [5], and a focused library for coronaviruses, including SARS-CoV-2 [6].

We and others have utilized PhIP-Seq to successfully identify novel autoantigens associated with autoimmune diseases [7–9], to broadly characterize allergy-related antibodies [4], to quantitatively compare the antibody repertoires of term and preterm neonates [10], to assess changes in the anti-viral antibody response after bone marrow transplant [11], to characterize the self-reactivity of broadly neutralizing HIV antibodies [12–14], to link enteroviral infection with acute flaccid myelitis [15], and for use in large cross-sectional and longitudinal studies of exposure and response to hundreds of human viruses and thousands of bacterial proteins in healthy individuals and in individuals infected with HIV or measles [3, 16–18]. In addition, we recently used PhIP-Seq to assess how antibody responses to endemic coronaviruses modulate COVID-19 convalescent plasma functionality [6] and evaluated the heritability of antibody responses [19].

The output from PhIP-Seq experiments are read count matrices, similar to RNA-Seq data, but important differences in the data structures, experimental design, and study objectives exist between the two sequencing-based methods. RNA-Seq experiments typically focus on differentially expressed genes or transcripts between experimental groups, rather than identifying expressed genes for any particular sample. The objective of PhIP-Seq experiments however is typically just that: detecting peptide antibody reactivity in an individual sample. Thus, in contrast to RNA-Seq experiments, the design of PhIP-Seq experiments requires the use of negative controls (i.e. “mock” immunoprecipitations (IPs) lacking antibody input, also referred to as beads-only samples), which are typically included as 4 to 8 wells of a 96-well plate. This generates a “n versus 1” mock IPs versus sample comparison, in contrast to the most common n_1_ versus n_2_ two-group comparison in RNA-Seq. In addition, genes with low read counts are presumed to have little biological relevance, and RNA-Seq data workflows typically filter out lowly expressed genes (measured as counts-per-million) prior to analysis. In PhIP-Seq experiments however, peptides with low read counts may have biological relevance and are not filtered out in advance. That said, under suitable assumptions, such as equality of variances in both groups, a two-group comparison with a single sample in one group can still be carried out.

Significant advances in normalization and analysis methods for RNA-Seq data have been made in recent years, with edgeR, DESeq2, and voom among the most popular open-source software packages available [20–23]. These methods model the number of reads using a negative binomial distribution to account for the inflated variance due to biological variability between samples in comparison to the expected variance of the binomial distribution. Parameter estimation is based on empirical Bayes methods to borrow strength across transcripts, stabilizing the estimates of the respective standard errors. Upregulated genes in RNA-Seq experiments draw a higher proportion of reads than expected for a given library size (here, total read counts), resulting in lower than expected read counts for other genes given that library size (“competing resources”). Thus, a normalization factor for each sample is calculated in RNA-Seq experiments to account for this effect [24]. One assumption is that the majority of genes are not differentially expressed when comparing cases to controls.

In this manuscript we investigated whether the publicly available method edgeR [20] for normalization and analysis of RNA-Seq data is also suitable for PhIP-Seq data. We highlight some of the differences between PhIP-Seq and RNA-Seq experiments and data sets, which motivates the development of a new methodology for PhIP-Seq data explicitly based on the assumed data generating mechanism, rather than adapting existing RNA-Seq approaches. To that end, we introduce a Bayesian framework specifically tailored for data from PhIP-Seq experiments (Bayesian Enrichment Estimation in R, BEER). Using simulation studies and data sets from existing HIV and SARS-CoV-2 studies, we investigate what improvements in sensitivity and specificity can be made, highlight the importance of empirical Bayes methods, and assess the effect of the number of mock IP samples on sensitivity and specificity.

## Results

**Fig. 1:**
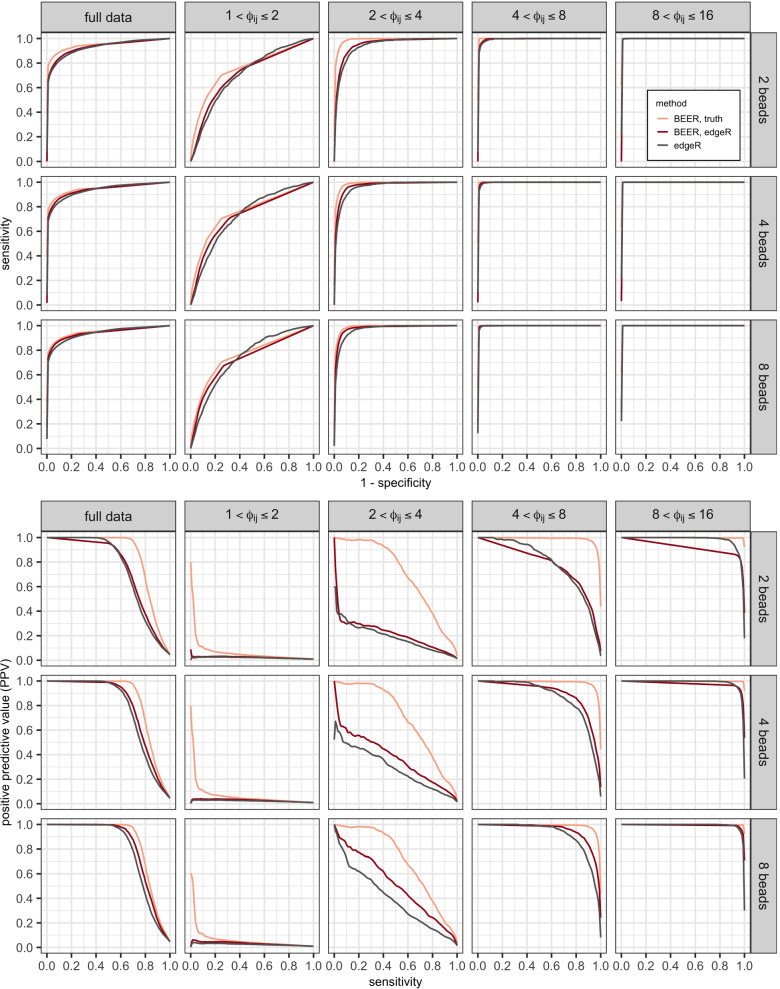
Average receiver operating characteristic (ROC; top panels) and precision-recall (PR; bottom panels) curves calculated from ten simulations, comparing edgeR (black lines) and BEER (red lines) across fold-change categories and number of beads-only samples available. Curves for BEER using the actual simulation shape parameters in the prior distributions (orange lines) are added to show the effect of sampling variability in these parameters. Results for fold changes above 16 are omitted since in all instances peptides were correctly classified as enriched

**Simulation.** Regardless of the approach used to estimate prior parameters, BEER has high discriminatory power for identifying enriched peptides (Fig. 1, Supplementary Fig. S1). In general, BEER using methods of moments (MOM) or maximum likelihood estimates (MLE) for the shape parameters in the beads-only prior distributions performed worse than BEER using edgeR parameter estimates, highlighting the importance of borrowing strength across peptides for improved parameter estimation (Supplementary Fig. S1, Table S1). The stability of parameter estimates also affected the improvement in BEER predictive performance by the number of beads-only samples used. While BEER with MOM and MLE parameter estimates greatly benefited from the inclusion of more beads-only samples in the experiment, BEER using edgeR parameter estimates had much less pronounced improvements as the number of beads-only samples was increased (Supplementary Figs. S2, S3, and Table S1). Using these edgeR parameter estimates, BEER posterior probabilities of enrichment were well-calibrated (Supplementary Fig. S4), and estimates of fold changes were accurate (Supplementary Fig. S5). Thus, we recommend the edgeR parameter estimates as default and imply their use when simply referring to BEER as the method used.

In general, performances of edgeR and BEER for identifying enriched peptides were surprisingly similar (Fig. 1, Supplementary Table S1). Both methods yielded near perfect receiver operating characteristic (ROC) curves for peptide fold changes above 4 (area under the curves (AUCs) > 0.99), and still outstanding ROC curves for fold changes between 2 and 4 (AUCs between 0.94 and 0.98) even when a design with only 2 beads-only samples was employed. Peptide fold changes less than 2 were harder to detect, reflected in substantially lower AUCs between 0.71 and 0.74. Precision-recall (PR) curves for edgeR and BEER are noticeably different for intermediate fold changes between 2 and 4, where also most improvement is (theoretically) possible for BEER if improved estimates of shape parameters used in the prior distributions were available. As expected from the near perfect ROC curves, reliable detection of peptides with fold changes above 4 is possible with a low rate of false positives (Fig. 1, Supplementary Table S2). For small fold changes less than 2 the positive predictive value (PPV) is generally poor, which is expected as the ROCs are modest and most peptides are not enriched.

**Fig. 2:**
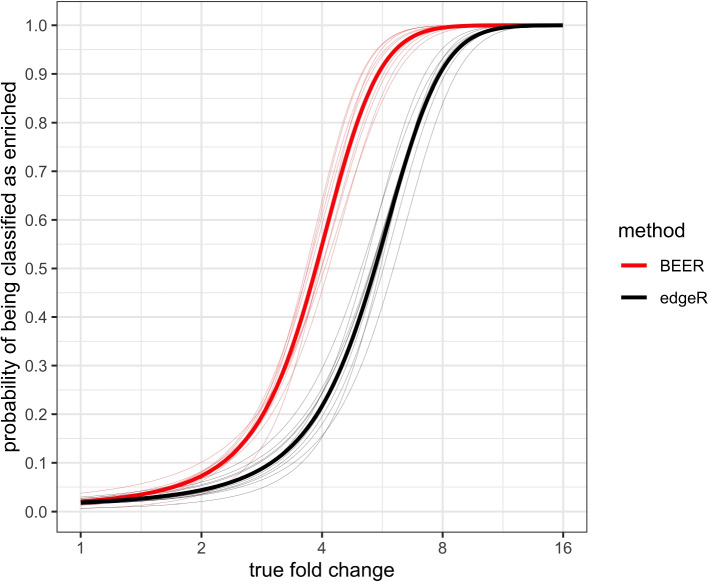
Estimated probabilities for correctly identifying enriched peptides (y-axis) as a function of the fold-change (x-axis) for each of ten simulated data sets based on logistic regression models. BEER posterior probability cut-offs were selected to achieve a false discovery rate of 5% in each simulation (see Section 4). Thin lines indicate the individual simulations, thick lines the respective averages

Under commonly employed false discovery rate (FDR) control, BEER has a higher probability of correctly identifying enriched peptides than edgeR across all fold-changes, and the difference in probability is most pronounced for moderate fold changes between 2 and 8 (Fig. 2). For example, under a FDR control of 5%, on average, the probability of identifying a peptide with a 4 fold change is 53% for BEER, but only 21% for edgeR. Similarly, BEER has a probability of at least 50% to detect fold changes above 3.7 under this FDR control, while edgeR requires a fold-change of at least 5.5. Of note, the BEER posterior probability cut-offs in the ten simulations to achieve a 5% FDR (see Section 4) were between 0.25 and 0.49; thus, *using a commonly employed posterior probability cut-off of 0.5 leads to fewer false positives on average*.

**Fig. 3:**
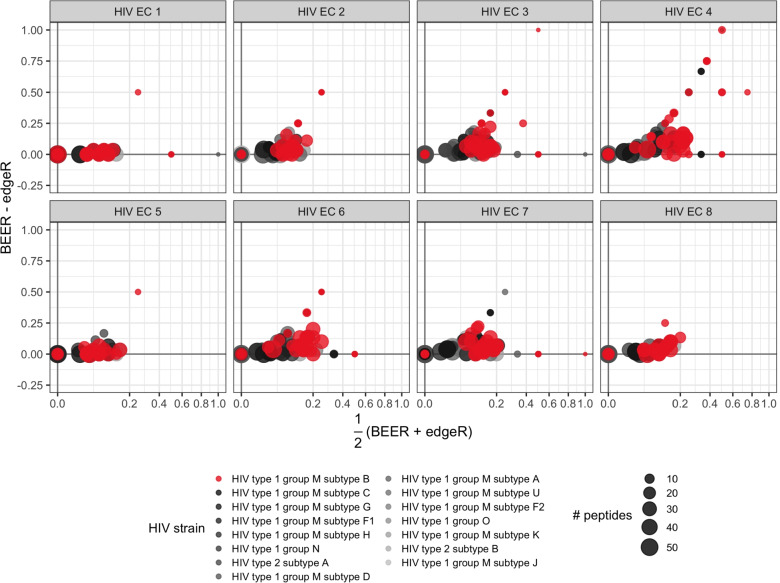
Bland-Altman (MA) plots for the proportion of enriched peptides by protein, for eight elite controller samples. Points represent individual proteins, point colors indicate protein virus types, point diameters indicate the number of peptides tiling the respective proteins. All subjects shown here were infected with subtype B (red)

**HIV elite controllers.** Both BEER and edgeR had no false positives across the six mock IP samples using a posterior probability cut-off of 0.5 for BEER and an FDR control of 5% for edgeR (corresponding to *p*-value cut-offs between $1.0 \times 10^{-3}$ and $2.4 \times 10^{-3}$ across the eight samples). As the non-replicated serum samples are from individuals infected with HIV subtype B, we expected stronger antibody reactivity to proteins from HIV subtype B, and indeed, BEER and edgeR detect more enrichments to peptides tiling proteins from HIV subtype B than proteins of any other HIV strain represented in the library (Fig. 3). Notably, for any particular subtype B protein, BEER detects more enriched peptides than edgeR (while expected to have a lower type I error with a posterior probability cut-off of 0.5, see above). Some antibody reactivity to proteins from other HIV subtypes is expected due to cross-reactivity (Supplementary Fig. S6).

BEER and edgeR detected enrichments generally agreed across two technical replicates of a sample from an HIV subtype A infected individual (Supplementary Fig. S7). Using the same cut-offs as above for declaring enrichment, both methods had high agreement (concordance about 0.90–0.95) between the two samples among the top 100 peptides ranked by posterior probabilities and *p*-values, respectively (Supplementary Fig. S8). This is not surprising as the BEER posterior probabilities for peptides ranked 100 in the two technical replicates were 0.99 and 1.00 respectively, and the edgeR *p*-values were $3.1 \times 10^{-4}$ and $9.4 \times 10^{-5}$ with respective 5% FDR cut-offs of $1.5 \times 10^{-3}$ and $1.8 \times 10^{-3}$, respectively. Thus, both methods exhibit high confidence that most of the peptides among the top 100 are truly enriched (Supplementary Fig. S9). While BEER concordance decreases but remains above 0.90 when considering the lists of peptides with ranks up to 200, the edgeR concordance does drop more noticeably (Supplementary Fig. S8), potentially indicating a higher sensitivity in BEER for peptides with smaller fold changes. Comparing subtype A peptides across technical replicates, BEER and edgeR had very similar performance. Compared to other subtypes, both methods also showed less discordance among subtype A peptides (Supplementary Table S3).

**CoronaScan.** In a round-robin, leaving one mock IP sample out in turn, no false positives (i.e., peptides falsely called enriched) were produced by BEER or edgeR across the eight mock IPs in the CoronaScan data using a posterior probability cutoff of 0.5 for BEER and an FDR control of 5% for edgeR (*p*-value cutoffs ranged from $3.3 \times 10^{-4}$ to $1.1 \times 10^{-3}$). Among the six serum samples from individuals prior to the COVID-19 pandemic, BEER and edgeR show more enrichment to peptides tiling human coronaviruses, but generally no enrichment of SARS-CoV-2 peptides (VRC 1 – VRC 6, Supplementary Fig. S10). In contrast, among the four samples from a single individual infected with SARS-CoV-2, taken at 10, 11, 12 and 13 days since symptom onset, an enrichment of SARS-CoV-2 protein tiling peptides is apparent. Particularly on day 13 after symptom onset, the patient presumably has produced a large number of antibodies which were detected by both BEER and edgeR. Of note, this was the first day the a SARS-CoV-2 antibody test was positive (D13, Supplementary Fig. S10), further demonstrating the power and utility of the PhIP-Seq approach.

Comparing peptide replicates in the CoronaScan library, concordance among the most enriched peptides (when ranked by posterior probabilities and *p*-values for BEER and edgeR, respectively) was generally above 0.80 (Supplementary Fig. S11). For example, in sample VRC 4 both methods show high confidence that the top 35 peptides are truly enriched (posterior probabilities of 0.97 and 1.00 for BEER, and *p*-values of $8.6 \times 10^{-5}$ and $1.9 \times 10^{-6}$ for edgeR, well below the 5% FDR cut-off derived *p*-value of $4.2 \times 10^{-4}$, Supplementary Fig. S12). BEER and edgeR perform very similarly in this example (with BEER slightly better between peptides 10 and 20), showing concordance above 0.80 before dropping significantly after about 50 peptides. The somewhat lower concordances compared to the same ranking metric derived from the two technical replicates in the HIV elite controllers above can possibly explained by the smaller range of proportions of reads pulled in the CoronaScan platform, and therefore higher correlation among these proportions comparing the technical replicates in the HIV data (Supplementary Fig. S8 left, versus Supplementary Fig. S11 left). Among all CoronaScan samples the overall concordance of peptide pair enrichment calls was outstanding, with less than 1% discordant calls in each sample, for both BEER using a posterior probability cutoff of 0.5 and 5% FDR cutoffs for edgeR (corresponding to *p*-value cutoffs between $3.3 \times 10^{-4}$ and $1.1 \times 10^{-3}$, Supplementary Table S4).

## Discussion

In this manuscript we investigated whether the publicly available method edgeR [20] for normalization and analysis of RNA-Seq data is also suitable for PhIP-Seq data. With the exception of calculating one-sided *p*-values to infer peptide reactivity, no “tweaks” were necessary in the implementation, and we found the approach to be effective. However, using simulation studies we showed that substantial improvements are possible with a Bayesian framework specifically tailored for data from PhIP-Seq experiments (Bayesian Enrichment Estimation in R, BEER). In particular for peptides showing weaker reactivity, we saw an improvement of sensitivity with lower false positive rates when standard cut-offs were employed (posterior probability > 0.5 for the Bayesian method and a Benjamini-Hochberg false discovery rate control of 5% for edgeR). This comparison might be perceived as somewhat unfair, as the data were simulated from a model similar to that underlying BEER, which we recognize. However, BEER was implemented in a way we believe reflects the true data generating mechanism, which is also corroborated by the posterior predictive assessment [25] of the HIV EC 1 data, as the observed read counts are well supported by the distributions derived under the model (Supplementary Fig. S13). BEER also showed better performance on real data such as the data from the HIV elite controllers, where BEER detected more enriched peptides of the correct HIV subtype than edgeR. This improved performance comes at a price of increased computational cost. While edgeR delivers almost instantaneous results, the Markov chains underlying BEER are time consuming. However, since laboratory prep and sequencing are expensive and certainly take more time than running such Markov chains, we believe utilizing extra CPU time to run BEER, as shown in our workflow (https://github.com/athchen/beer_manuscript), may yield worthwhile additional discoveries.

It was initially surprising to us how well edgeR fared on PhIP-Seq data despite being designed specifically for RNA-Seq data. While important differences between PhIP-Seq and RNA-Seq data structures exist, as previously described, edgeR captures some of the most important effects that exist in both types of data. For example, unlike RNA-Seq, the PhIP-Seq experimental protocol requires the use of negative controls (i.e. samples with no serum) on a 96-well plate. The observed read counts mapped to the peptides among those negative controls show a very strong peptide-dependent bias in library representation and/or “background” binding to the beads, such that some peptides consistently draw a much higher proportion of reads than others (Supplementary Fig. S14). However, in a “n versus 1” mock IPs versus sample comparison where inference is drawn for each peptide, these differences among peptides are similar to the biological variability observed between genes in RNA-Seq [26]. In addition, edgeR models read counts using a negative binomial distribution to account for larger than binomial variability between samples, an effect we also observe in PhIP-Seq data (Supplementary Fig. S15). And while we expect reactive peptides in a serum sample to pull a large number of reads, and thus – after adjusting for library size – expect non-reactive peptides in a serum-sample to have fewer reads on average than the corresponding beads-only sample peptides (Supplementary Fig. S16), the resulting attenuation constant in essence is the same as the scale factors derived from the trimmed mean of M-values approach in edgeR [20, 24].

Our findings also highlight the importance of empirical Bayes methods for parameter estimation. Methods of moments and maximum likelihood estimates for individual peptide prior distribution shape parameters performed substantially worse than those obtained by borrowing strength across peptides. By also using the true shape parameters in our simulations to assess sensitivity and specificity, we were able to demonstrate that, particularly for intermediate fold changes, better performance could be achieved by improving procedures to estimate these parameters. Our findings also give guidance for experimental design, such as the chosen number of mock IPs per 96-well plate. Allocating more beads-only samples to a plate improves estimation of these shape parameters that largely quantify between sample variability of the probabilities of a specific peptide to pull a read. Choosing more beads-only samples means reduced number of biological samples assayed per plate, which for the practitioner means additional cost and labor for more plates. In previous experiments, the number of mock IPs per plate was typically between 4 and 8. Our simulation studies showed that this is appropriate, as the observed difference in performance between 8 and 4 beads-only samples was much less than the observed difference between 4 and 2 mock IPs, indicating diminishing returns.

A few technical details should also be discussed further. As described in the Section 4, reads from highly reactive peptides (initial fold change estimate above 15) are removed from the data of mock IPs and the actual sample before BEER analysis, and the respective library sizes are recalculated. The main reason for doing so is simply to stabilize the inference and improve scalabilty, as allowing for extreme fold changes in the Bayesian model for a few peptides can affect these features. We verified that there were “no false positives” in the sense that all posterior probabilities for these highly reactive peptides were 1 if not excluded from the analyses. Thus, the chosen “highly enriched” threshold of 15 is likely conservative. We also note that the Bayesian model can be extended to run Markov chains for multiple samples against the beads-only samples. However, the resulting increase in parameter space makes this a challenging endeavor, especially with regards to scalability. It could be argued that for the same reasons stated above an increase in CPU time should be acceptable if this leads to an improvement detecting reactive peptides. However, we did not observe an improvement in detecting antibody reactivities in simulation studies we performed (data now shown). No notable improvements were observed when the same peptide was simulated as enriched in all samples compared to the beads-only, and a deterioration was observed when reactivity was not common to all peptides. Since in real life experiments we seldom expect the exact same peptides to be reactive, we did not pursue this line of research further.

In summary, antibodies commonly serve as indicators of environmental exposures and immune responses, and Phage ImmunoPrecipitation Sequencing allows for quantification of antibody binding to hundreds of thousands of peptides, in individuals and large cohorts. We believe that this technology will play an even more prominent role in the future, addressing questions about exposures and health outcomes in populations, as well as individualized medicine. In this manuscript, we introduce a method and a software package for analyzing data from this technology, contrast it with an existing RNA-Seq software package that can be retooled for PhIP-Seq data, and share a workflow with practitioners to successfully carry out their own analyses of data resulting from PhIP-Seq experiments.

## Methods

**A Bayesian model for detecting antibody enrichment.** A succinct summary of the model notation is provided in Supplementary Table S5. On a 96-well plate suppose we observe $Y_{ij}$ read counts for peptide $i \in \{1, 2, \ldots , P\}$ in sample $j \in \{1, 2, \ldots , 96\}$. Let $n_{j} = \sum _{i} Y_{ij}$ denote the total read count (library size) for sample *j*. Without loss of generality, assume samples $\{1, 2, \ldots , N\}$ are mock IP (beads-only) samples. To infer reactivity, we compare one sample to all beads-only samples on the same plate. Our hierarchical model to infer peptide reactivity in a sample $j \in \{N+1, \ldots , 96\}$ is described as follows.

$$\begin{aligned} Y_{ij}|\theta _{ij}&\sim \text {Binomial}(n_{j}, \theta _{ij}) \\ \theta _{ij}|a_{i0}, b_{i0}, c_{j}, \phi _{ij}&\sim \text {Beta}(f_{a}(c_{j} \phi _{ij} \mu _{i0}, \sigma ^{2}_{i0}), f_{b}(c_{j} \phi _{ij} \mu _{i0}, \sigma ^{2}_{i0})) \\ c_{j}&\sim \text {Beta}(a_{c}, b_{c}) \\ \phi _{ij}|Z_{ij}&\sim (1 - Z_{ij}) \cdot 1 + Z_{ij}(\phi _{min} + \text {Gamma}(a_{\phi }, b_{\phi })) \\ Z_{ij}|\pi _{j}&\sim \text {Bernoulli}(\pi _{j})\\ \pi _{j}&\sim \text {Beta}(a_{\pi }, b_{\pi }) \end{aligned}$$

The main parameter of interest $Z_{ij}$ is a binary indicator denoting whether peptide *i* elicits an enriched antibody response in sample *j* (a 0 indicates no, a 1 indicates yes). The prior is a Bernoulli with success probability $\pi _j$. For all mock IP samples, this success probability is zero. For sample *j*, $\pi _{j}$ is modeled as a beta distribution. The shape parameters $a_{\pi }$ and $b_{\pi }$ of the Beta prior distributions are chosen as 2 and 300 in our applications, to reflect peptide enrichment seen in previous studies, but also make it sufficiently diffuse to support a range of proportions (Supplementary Fig. S17). The parameter $\phi _{ij}$ is the fold change observed for peptide *i* in sample *j*. It is equal to 1 if $Z_{ij} = 0$, i.e., peptide *i* does not elicit an enriched antibody response in sample *j*. Enriched peptides are expected to pull a larger proportion of reads, so only fold changes larger than 1 are considered. Here, we model the fold-change as a shifted gamma distribution (with shape parameters $a_{\phi }$ = 1.25 and $b_{\phi }$ = 0.1, Supplementary Fig. S17), with the magnitude of the shift $\phi _{min}$ being the minimum fold-change assumed for an enriched peptide (chosen as 1 in our applications). In the presence of reactive peptides pulling reads, non-reactive peptides in sample *j* will have less reads than expected from the beads-only samples where no reactive peptides exist by definition. We denote this attenuation constant for sample *j*, which is similar to the trimmed mean of M-values (TMM) scale factor used in edgeR [24], with $c_{j}$. Typically, only a minority of peptides in a sample show reactivity and the attenuation constant usually is between 0.5 and 1 (being equal to 1 in mock IP samples). In this application, we chose a Beta prior with scaling constants $a_{c} = 80$ and $b_{c} = 20$ (Supplementary Fig. S17; the attenuation constant is equal to 1 in the mock IP samples). The observed read counts $Y_{ij}$ are modeled using a Binomial$(n_{j}, \theta _{ij})$ distribution, where $\theta _{ij}$ denotes the probability that peptide *i* pulls a read in sample *j*, and $n_{j}$ denotes the total library size in sample *j*. This Binomial probability is modeled using a Beta prior distribution, and the shape parameters depend on the expected peptide read counts observed in the mock IP samples (estimation procedures described below), the fold change $\phi _{ij}$, and the attenuation constant based on the reads pulled by all reactive peptides in the sample.

**Shape parameter estimation.** We define two functions, $f_{a}$ and $f_{b}$, used for the description of the Beta shape parameters *a* and *b* given a mean $\mu$ and a variance $\sigma ^{2}$.

$$\begin{aligned} f_{a}(\mu , \sigma ^{2}) = \frac{\mu ^{2}(1 - \mu )}{ \sigma ^{2}} - \mu \quad \text {and} \quad f_{b}(\mu , \sigma ^{2}) = f_{a}(\mu , \sigma ^{2}) \left( \frac{1}{\mu } - 1\right) . \end{aligned}$$

The parameterization for the Beta shape parameters above is the same as used in the methods of moments estimation, and the mean and variance for peptide *i* in a beads-only sample (e.g., for a Beta distribution with shape parameters $a_{i0}$ and $b_{i0}$) are given by

$$\begin{aligned} \mu _{i0} = \frac{a_{i0}}{a_{i0} + b_{i0}} \quad \text {and} \quad \sigma ^{2}_{i0} = \frac{a_{i0}b_{i0}}{(a_{i0} + b_{i0})^{2}(a_{i0} + b_{i0} + 1)}. \end{aligned}$$

Since each sample generally contains more than a million of reads, estimates of the Binomial probabilities $\hat{\theta }_{ij} = \frac{Y_{ij}}{n_{j}}$ in the mock IP samples $j \in \{1, 2, \ldots , N\}$ are very precise. Method of moments (MOM) estimates for the peptide *i* shape parameters $a_{i0}$ and $b_{i0}$ can be derived by equating the mean and variance of the above Binomial estimates across all beads-only samples to the mean and variance of the Beta$(a_{i0}, b_{i0})$ distribution.

$$\begin{aligned} \hat{\theta }_{i0}= & {} \frac{1}{N} \sum\limits_{j = 1}^{N} \hat{\theta }_{ij} \\ \hat{\sigma }^{2}_{i0}= & {} \frac{1}{N - 1} \sum\limits_{j = 1}^{N} (\hat{\theta }_{ij} - \hat{\theta }_{i0})^{2}. \end{aligned}$$

The MOM estimates for $a_{i0}$ and $b_{i0}$ are then given by

$$\begin{aligned} \hat{a}_{i0}^{\text {MOM}}= & {} f_{a}(\hat{\theta }_{i0}, \hat{\sigma }^{2}_{i0})\\ \hat{b}_{i0}^{\text {MOM}}= & {} f_{b}(\hat{\theta }_{i0}, \hat{\sigma }^{2}_{i0}). \end{aligned}$$

Maximum likelihood estimates (MLEs) for $a_{i0}$ and $b_{i0}$ were derived using the Broyden, Fletcher, Goldfarb and Shanno quasi-Newton optimiziation algorithm with box constraints [27], as implemented in the R optim() function.

Numerous papers have demonstrated the benefits of shrinkage or variance stabilization in high throughput genomics experiments, borrowing strength across units such as genes and proteins [20, 22, 28, 29]. This can be particularly important when the sample sizes are small, such as the number of mock IP experiments on each plate in our application, but neither the MLEs nor the MOM estimates described above make use of this. In contrast for example, edgeR uses an emprical Bayes approach [30] to approximate the larger than binomial variability observed in the RNA-Seq read counts, and to stabilize these variance estimates, which are characterized by the tagwise dispersion parameter (the squared coefficient of variation of $\hat{\theta }_{ij}$, denoted here as $\tau ^{\text {edgeR}}_{ij}$) [20]. We note that we can use the estimates of these tagwise dispersion parameters to derive new estimates of the variances for our Binomial probabilities $\theta _{ij}$. Specifically, for peptide *i* we have $\left( \hat{\sigma }^{\text {edgeR}}_{i0}\right) ^{2} = \tau ^{\text {edgeR}}_{ij}*\hat{\theta }_{i0}^{2}$, and thus

$$\begin{aligned} \hat{a}_{i0}^{\text {edgeR}}= & {} f_{a}\left( \hat{\theta }_{i0}, \left( \hat{\sigma }^{\text {edgeR}}_{i0}\right) ^{2}\right) \\ \hat{b}_{i0}^{\text {edgeR}}= & {} f_{b}\left( \hat{\theta }_{i0}, \left( \hat{\sigma }^{\text {edgeR}}_{i0}\right) ^{2}\right) \end{aligned}$$

The Beta parameters *a* and *b* can be thought of as the number of successes and the number of failures, respectively, in $a + b$ trials. The Markov Chain Monte Carlo (MCMC) sampler Just Another Gibbs Sampler (JAGS) can encounter numerical issues when either of those is less than 1. In PhIP-Seq experiments *a* is much smaller than *b* as a peptide only pulls a fraction of the total number of reads even when reactive. Thus, to avoid these numerical problems, we set *a* to be the larger number of the estimated value and 1, and then estimate *b*.

**Markov chain Monte Carlo.** The model was implemented in JAGS (4.3.0) and run using the R interface for JAGS, rjags [31–33]. JAGS is a Gibbs sampler based on slice sampling as decribed in Neal (2003) [34]. We use maximum likelihood estimates to select starting values of $\theta _{ij}, Z_{ij}, \phi _{ij}, c_{j}$, and $\pi _{j}$ to initialize the Markov chain for non beads-only sample *j*. As described above, $\hat{\theta }_{ij}^{\text {init}} = \hat{\theta }_{ij}$ is the MLE of the binomial probability calculated from the read counts. Since $Z_{ij}$ is needed to update $c_{j}, \pi _{j}, \phi _{ij}$, we set $\hat{Z}_{ij}^{\text {init}}$ = 1 if its observed read count is at least twice as large as the expected read count in a beads-only sample. That is, $\hat{Z}_{ij}^{\text {init}}$ = 1 if $Y_{ij} \ge 2 n_{j} \hat{\theta }_{i0}$, and 0 otherwise. The initial value for the attenuation constant is derived by regressing the observed read counts on the expected reads count for all non-enriched peptides in that sample, with $\hat{c}_{j}^{\text {init}}$ being the slope estimate

$$\begin{aligned} \hat{c}_{j}^{\text {init}} = \frac{\sum _{i=1}^{P} (1 - \hat{Z}^{\text {init}}_{ij}) \times Y_{ij} \times n_{j} \times \hat{\theta }_{i0}}{\sum _{j=1}^{P} (1 - \hat{Z}^{\text {init}}_{ij}) \times (n_{j} \hat{\theta }_{i0})^{2}}. \end{aligned}$$

The initial value for the proportion of enriched peptides is the average of all enrichment indicators

$$\begin{aligned} \hat{\pi }_{j}^{\text {init}} = \frac{1}{P}\sum\limits_{i=1}^{P} \hat{Z}_{ij}^{\text {init}}, \end{aligned}$$

and the respective peptide fold changes are initialized as

$$\begin{aligned} \hat{\phi }_{ij}^{\text {init}} = (1 - \hat{Z}_{ij}^{\text {init}}) + \hat{Z}_{ij}^{\text {init}} \frac{Y_{ij}}{n_{j} \times \hat{c}_{j}^{\text {init}} \times \hat{\theta }_{i0}}. \end{aligned}$$

Since $c_{j}$ and $\pi _{j}$ are modeled using Beta distributions with no support at values 0 and 1, we use a small offset in the event that $c_{j}^{\text {init}} = 1$ and $\pi _{j}^{\text {init}} = 0$.

In PhIP-Seq experiments we commonly observe very reactive peptides [17, 35]. Allowing for extreme fold changes in the Bayesian model for a few peptides can affect the inference for other less reactive peptides, and can have negative consequences for numerical stability and scalability. In our applications, clearly enriched peptides defined as $\hat{\phi }^{\text {init}}_{ij}>$ 15 were filtered out before starting the Markov chain. Reads from such peptides in the mock IP and actual samples were removed, and the library sizes were recalculated.

**Peptide reactivity detection with edgeR.** To identify reactive peptides, each serum sample is compared to all beads-only samples from the same plate. Differential expression in edgeR is assessed for each unit (here, each peptide) using an exact test analogous to Fisher’s comparing means between two groups of negative binomial random variables, but adapted for overdispersed data [36]. Two-sided *p*-values were subsequently converted to one-sided *p*-values as the alternative to the null of no reactivity (fold change = 1) is reactivity, leading to read count enrichment and thus, fold-changes larger than 1. Multiple comparisons corrections were based on the Benjamini-Hochberg procedure, using false discovery rates to delineate enrichment across all peptides.

**Simulation study.** We simulated ten data sets based on the read counts observed in the HIV elite controller data described below. Each of these data sets had eight beads-only samples and twelve simulated serum samples. The twelve samples contain one beads-only sample run as an actual sample and two technical replicates (samples generated from the same parameters). For each simulated serum sample, we randomly selected 50 peptides as reactive. Among those, 10 peptides each had fold changes between 1 and 2, between 2 and 4, between 4 and 8, between 8 and 16, and between 16 and 32. Each data set was analyzed using the first two, four, and all eight beads-only samples to assess the sensitivity of the results to the number of beads-only used for analysis. For each data set and number of beads-only sample combination, we ran BEER with the true beads-only Beta $a_{0}, b_{0}$ prior parameters, estimated beads-only parameters using maximum likelihood, method of moments, and edgeR derived estimates.

Performance was primarily assessed using ROC and PR curves on the full data and fold-change subsets of the data. For each fold-change bin, curves were generated using all non-enriched peptides and enriched peptides within the specified fold-change group from the simulated serum samples (no peptides from beads-only samples were included). To ensure that all curves had the same support points, we used linear interpolation to approximate the sensitivity or positive predictive value respectively at each support point for each simulation. ROC curves started at 0 sensitivity and 0 false-positive rate, while PRC curves started at 0 sensitivity and perfect positive-predictive value. The interpolated curves were averaged point-wise to generate an average curve for each condition. The area under each ROC curve was calculated using trapezoidal approximation from the interpolated data points. We also used logistic regression to model the probability of identifying and enriched peptide by fold-change in each data set. Multiple comparisons for edgeR *p*-values were addressed using the Benjamini-Hochberg procedure to ensure a 5% FDR. Cut-offs for the posterior probabilities were selected in each data set to achieve 5% false positive calls.

**Examples.** Antibody reactivity counts for eight plates of data were generated using the PhIP-Seq assay and the VirScan library on serum samples from HIV elite controllers with HIV subtype A and B infections, and analyzed by Kammers et al. [14] to assess antibody profiles in HIV controllers and persons with treatment-induced viral suppression. We used count data for the 3,395 phage-displayed peptides spanning the HIV proteome in the VirScan library for ten samples and six-beads-only samples from one plate of data. Two of the ten samples are identical, run in duplicate on the same plate. To quantify the false-positive rate of each algorithm, we also ran each beads-only sample against the remaining five-beads-only samples in a round-robin.

The CoronaScan data consists of counts for 6,932 peptides for 10 serum samples and 8 beads-only samples from one plate of data [6]. Among the ten samples, six were pre-pandemic samples and four samples were from one individual infected with SARS-CoV-2. Samples from this individual were collected on days 10 through 13 since symptom onset. By design, each peptide is present in duplicate in the CoronaScan library, enabling us to assess the concordance of the fold-change estimates and the enrichment status within samples. We again ran each beads-only sample against the remaining 7 beads-only samples to assess false positive rates.

The example in the Discussion to highlight the strong peptide-dependent background binding to the beads was from a previous study to evaluates HIV antibody responses and their evolution during the course of HIV infection [17] and to generate a classifier for recent HIV infections [35].

## Supplementary Information

**Additional file 1**: Supplementary Materials.

## Data Availability

The data and code for the figures, tables, and benchmarks are freely available at https://github.com/athchen/beer_manuscript to ensure the reproducibility of our results. BEER can be run using the R package beer which is available on Bioconductor at https://bioconductor.org/packages/devel/bioc/html/beer.html.
