# Supplementary material for: Detecting antibody reactivities in Phage ImmunoPrecipitation Sequencing data
Source: BMC Genomics. 2022 Sep 15;23:654. doi: 10.1186/s12864-022-08869-y (PMC9476399; doi:10.1186/s12864-022-08869-y)
Supplement: Supplementary file 1 — Additional file 1: Supplementary Materials. [file 12864_2022_8869_MOESM1_ESM.pdf]

Supplementary Materials

	2 beads-only	4 beads-only	8 beads-only
**BEER, MOM**	0.884	0.939	0.944
$1 < \phi_{ij} \leq 2$	0.695	0.757	0.756
$2 < \phi_{ij} \leq 4$	0.881	0.959	0.975
$4 < \phi_{ij} \leq 8$	0.937	0.989	0.994
$8 < \phi_{ij} \leq 16$	0.953	0.994	0.995
**BEER, MLE**	0.886	0.939	0.944
$1 < \phi_{ij} \leq 2$	0.697	0.759	0.762
$2 < \phi_{ij} \leq 4$	0.886	0.959	0.976
$4 < \phi_{ij} \leq 8$	0.940	0.989	0.994
$8 < \phi_{ij} \leq 16$	0.953	0.994	0.996
**BEER, edgeR**	0.929	0.936	0.939
$1 < \phi_{ij} \leq 2$	0.712	0.727	0.728
$2 < \phi_{ij} \leq 4$	0.951	0.970	0.977
$4 < \phi_{ij} \leq 8$	0.992	0.994	0.996
$8 < \phi_{ij} \leq 16$	0.995	0.995	0.996
**edgeR**	0.926	0.934	0.936
$1 < \phi_{ij} \leq 2$	0.709	0.728	0.737
$2 < \phi_{ij} \leq 4$	0.940	0.960	0.967
$4 < \phi_{ij} \leq 8$	0.991	0.994	0.995
$8 < \phi_{ij} \leq 16$	0.996	0.996	0.996

Table S1: Area under the ROC curves shown in Figure 1. Both BEER using edgeR parameter estimates and edgeR had near perfect classification for peptides with fold-changes above 4, even when only four beads-only samples were used to estimate a_{i0} and b_{i0}.

2 beads-only samples				
	50% sensitivity	75% sensitivity	90% sensitivity	95% sensitivity
**BEER, edgeR**	0.953	0.501	0.173	0.099
$1 < \phi_{ij} \leq 2$	0.026	0.017	0.013	0.011
$2 < \phi_{ij} \leq 4$	0.217	0.125	0.07	0.047
$4 < \phi_{ij} \leq 8$	0.844	0.693	0.482	0.322
$8 < \phi_{ij} \leq 16$	0.926	0.888	0.866	0.846
**edgeR**	0.965	0.442	0.152	0.092
$1 < \phi_{ij} \leq 2$	0.023	0.016	0.013	0.011
$2 < \phi_{ij} \leq 4$	0.182	0.097	0.056	0.037
$4 < \phi_{ij} \leq 8$	0.881	0.674	0.432	0.245
$8 < \phi_{ij} \leq 16$	1.000	0.996	0.958	0.874

4 beads-only samples				
	50% sensitivity	75% sensitivity	90% sensitivity	95% sensitivity
**BEER, edgeR**	0.989	0.632	0.208	0.114
$1 < \phi_{ij} \leq 2$	0.031	0.020	0.014	0.012
$2 < \phi_{ij} \leq 4$	0.388	0.207	0.116	0.080
$4 < \phi_{ij} \leq 8$	0.957	0.887	0.698	0.513
$8 < \phi_{ij} \leq 16$	0.989	0.971	0.965	0.947
**edgeR**	0.988	0.534	0.168	0.102
$1 < \phi_{ij} \leq 2$	0.024	0.018	0.013	0.012
$2 < \phi_{ij} \leq 4$	0.281	0.142	0.075	0.055
$4 < \phi_{ij} \leq 8$	0.958	0.824	0.586	0.368
$8 < \phi_{ij} \leq 16$	1.000	0.999	0.978	0.936

8 beads-only samples				
	50% sensitivity	75% sensitivity	90% sensitivity	95% sensitivity
**BEER, edgeR**	0.997	0.728	0.222	0.121
$1 < \phi_{ij} \leq 2$	0.032	0.021	0.014	0.012
$2 < \phi_{ij} \leq 4$	0.508	0.282	0.154	0.111
$4 < \phi_{ij} \leq 8$	0.988	0.959	0.835	0.716
$8 < \phi_{ij} \leq 16$	0.995	0.993	0.992	0.984
**edgeR**	0.997	0.606	0.177	0.102
$1 < \phi_{ij} \leq 2$	0.025	0.018	0.014	0.012
$2 < \phi_{ij} \leq 4$	0.366	0.181	0.089	0.067
$4 < \phi_{ij} \leq 8$	0.990	0.910	0.694	0.523
$8 < \phi_{ij} \leq 16$	1.000	1.000	0.992	0.968

Table S2: Average positive predictive values for select sensitivities for the curves in Figure 1.

	BEER		edgeR	
Concordance	n	p	n	p
Subtype A				
Both enriched	16	0.078	14	0.069
Both not enriched	186	0.912	188	0.922
Discordant	2	0.001	2	0.001
Other Subtypes				
Both enriched	118	0.037	94	0.029
Both not enriched	3039	0.952	3085	0.967
Discordant	34	0.011	12	0.004

Table S3: Concordance of enrichment calls between two technical replicates of an HIV subtype A infected individual for BEER and edgeR. A total of 204 peptides from subtype A and 3,191 peptides from other subtypes were present on the platform. n: number of peptides; p: proportion of peptides.

	BEER		edgeR	
Sample	n	p	n	p
VRC 1	3452	0.996	3454	0.997
VRC 2	3443	0.993	3443	0.993
VRC 3	3430	0.990	3423	0.988
VRC 4	3432	0.990	3427	0.989
VRC 5	3445	0.994	3444	0.994
VRC 6	3435	0.991	3433	0.990
SARS-CoV-2, D10 Ab-	3455	0.997	3450	0.995
SARS-CoV-2, D11 Ab-	3450	0.995	3446	0.994
SARS-CoV-2, D12 Ab-	3441	0.993	3439	0.992
SARS-CoV-2, D13 Ab+	3441	0.993	3437	0.992

Table S4: Concordance of enrichment calls between peptide pairs for all CoronaScan samples. Each sample has 3,366 unique peptide pairs. n: number of pairs with concordant enrichment calls; p: proportion of pairs with concordant enrichment calls.

$i \in \{1, 2, \cdots, P\}$	The peptide index.
$j \in \{1, 2, \cdots, 96\}$	The sample index.
Y_{ij}	The observed number of reads mapped to peptide i in sample j.
$n_j = \sum_{i=1}^{P} Y_{ij}$	The library size of sample j.
θ_{ij}	The probability that peptide i in sample j pulls a read.
Z_{ij}	The indicator whether peptide i in sample j is enriched (reactive).
π_j	The proportion of enriched peptides in sample j.
c_j	The attenuation constant for sample j.
ϕ_{ij}	The fold change of peptide i in sample j compared to the beads-only samples.
ϕ_{min}	The minimum fold-change for an enriched peptide (defined a priori).
a_{ij}, b_{ij}	The shape parameters for the prior distribution of θ_{ij} for peptide i in sample j.
a_π, b_π	The shape parameters for the prior distribution of the π_j.
a_c, b_c	The shape parameters for the prior distribution of the c_j.
a_ϕ, b_ϕ	The shape and scale parameters for the prior distribution of $\phi_{ij} \vert Z_{ij} = 1$

Table S5: The notation used in the BEER model. Parameters specific to beads-only samples are denoted with the subscript $i0$ (e.g. $a_{i0}, b_{i0}, \theta_{i0}$, etc.).

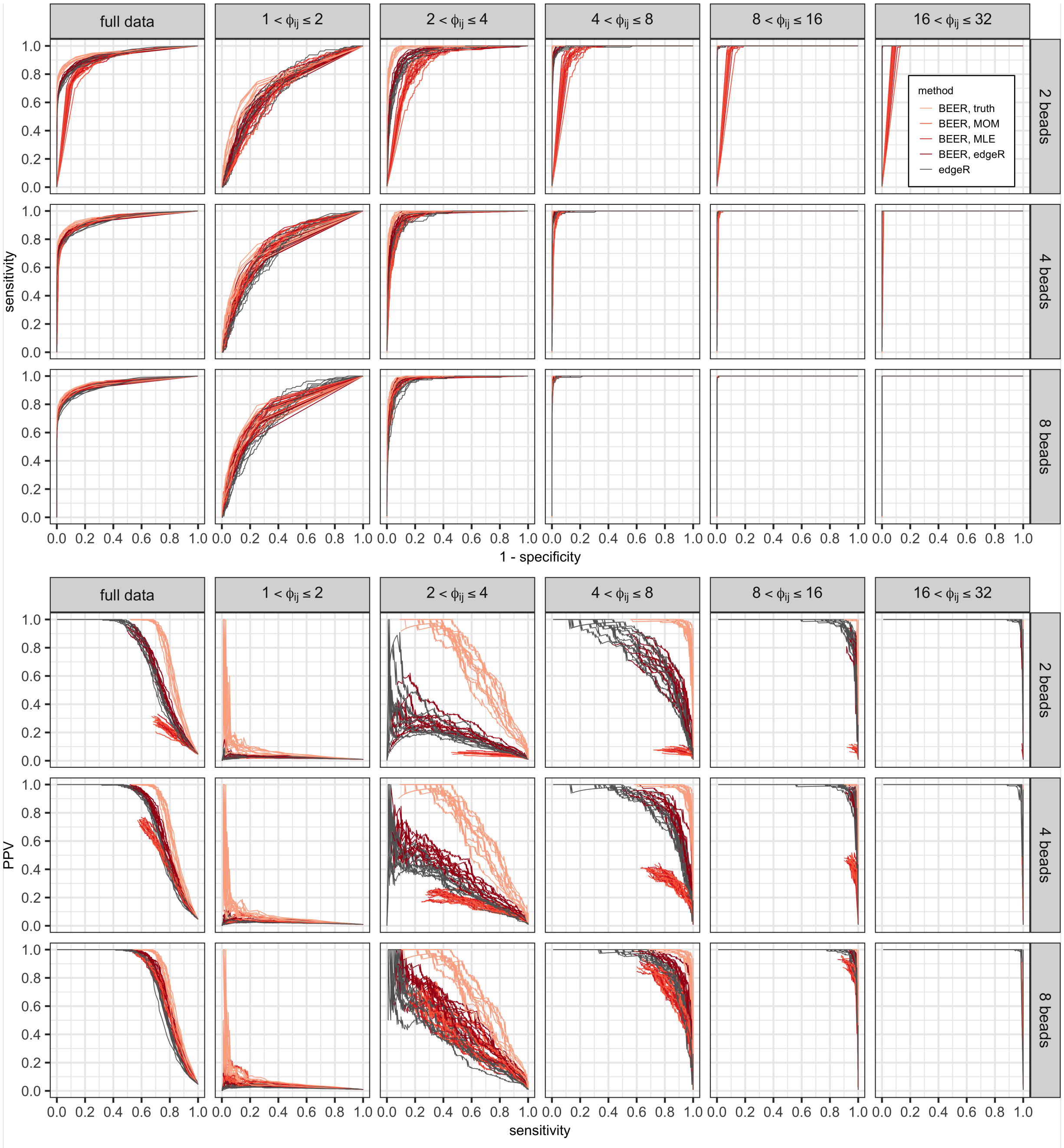

Figure S1: ROC (top panels) and PR (bottom panels) curves for various fold-change bins, by approach and method of estimation for a_{i0} and b_{i0}. The ROC and PR curves are generated by choosing a sliding set of p-value (edgeR) or posterior probability (BEER) cutoffs. This can induce a lower limit for the BEER sensitivity, since highly reactive peptides can achieve a posterior probability of one. For example, if half of the reactive peptides achieve a posterior probability of one, the sensitivity can never be lower than 0.5. This lower limit for indivual simulations was visualized in the PR curves.

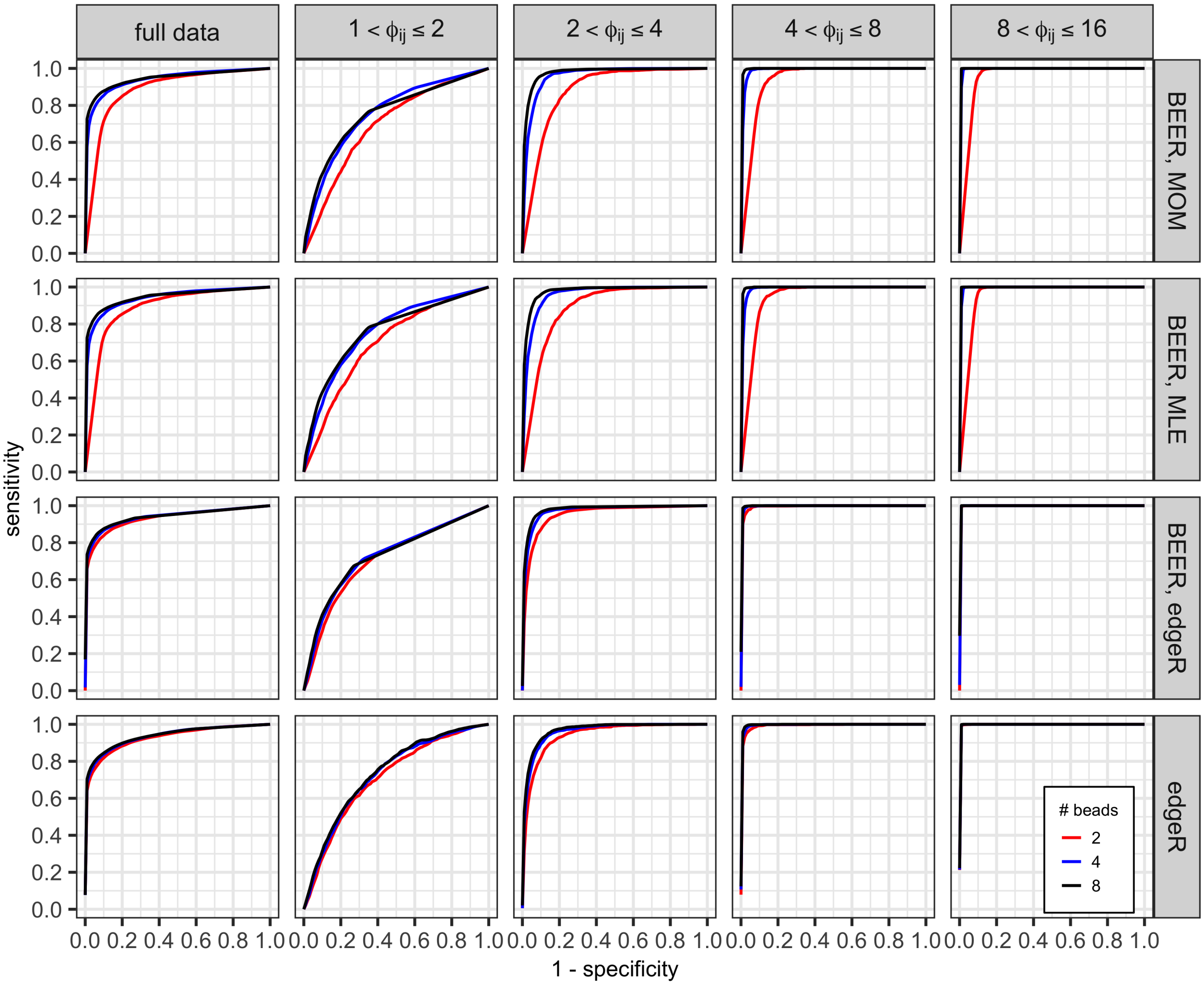

Figure S2: Averaged ROC curves comparing the performance of each method using 2 (red), 4 (blue), and all 8 (black) beads-only samples. Since there can be a lower limit on the BEER sensitivity (see the Figure S1 legend) the point on each ROC curve corresponding to the cutoff with the lowest sensitivity connects to the lower left (100% specificity and 0% sensitivity) and averages were calculated using these curves. Thus, the ROC curves should be interpreted with caution for these lower sensitivity segments.

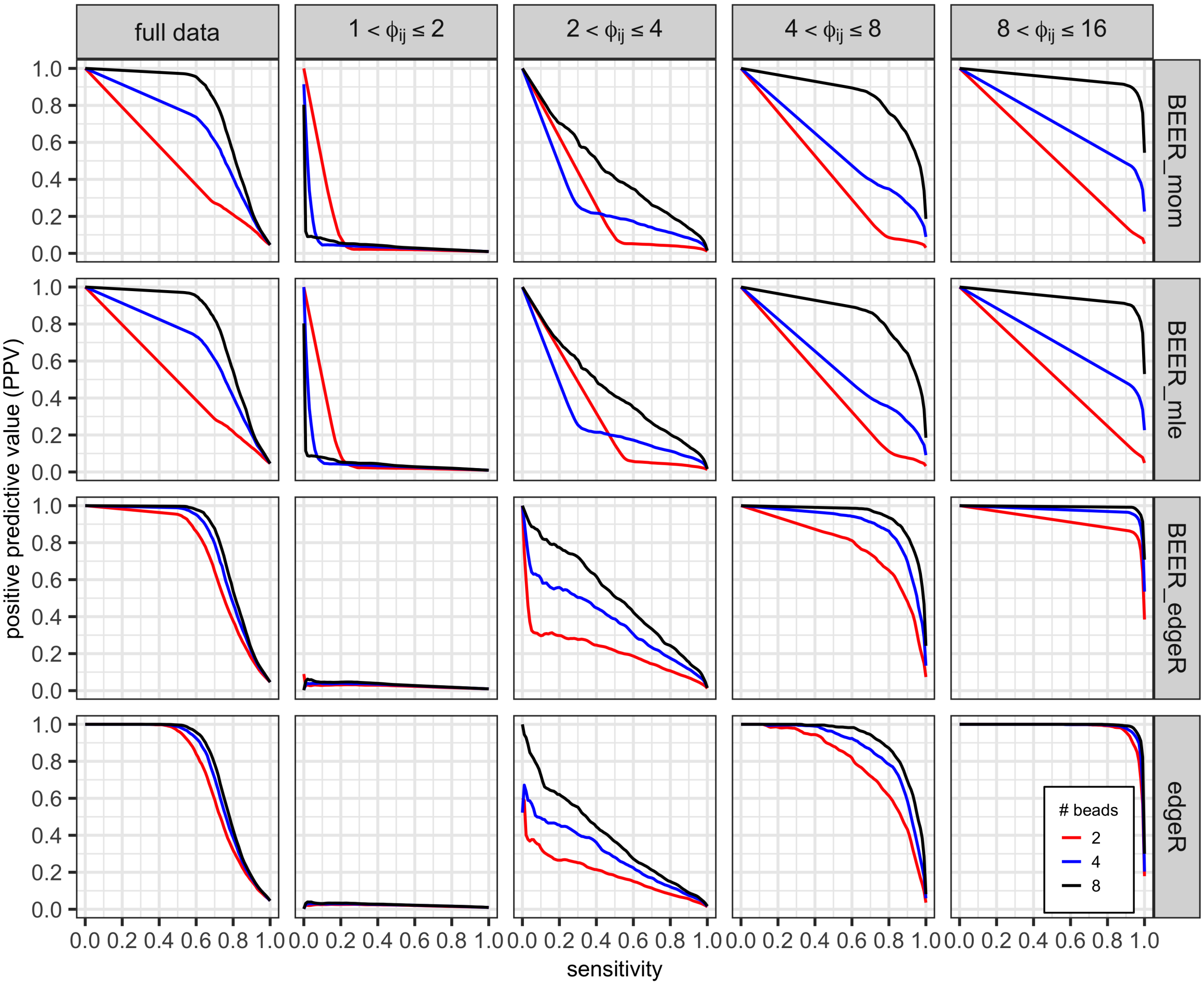

Figure S3: Averaged PR curves comparing the performance of each method using 2 (red), 4 (blue), and all 8 (black) beads-only samples. Since there can be a lower limit on the BEER sensitivity (see the Figure S1 legend) the point on each PR curve corresponding to the cutoff with the lowest sensitivity connects to the upper left (0% sensitivity and 100% positive predictive value) and averages were calculated using these curves. Thus, the PR curves should be interpreted with caution for these lower sensitivity segments.

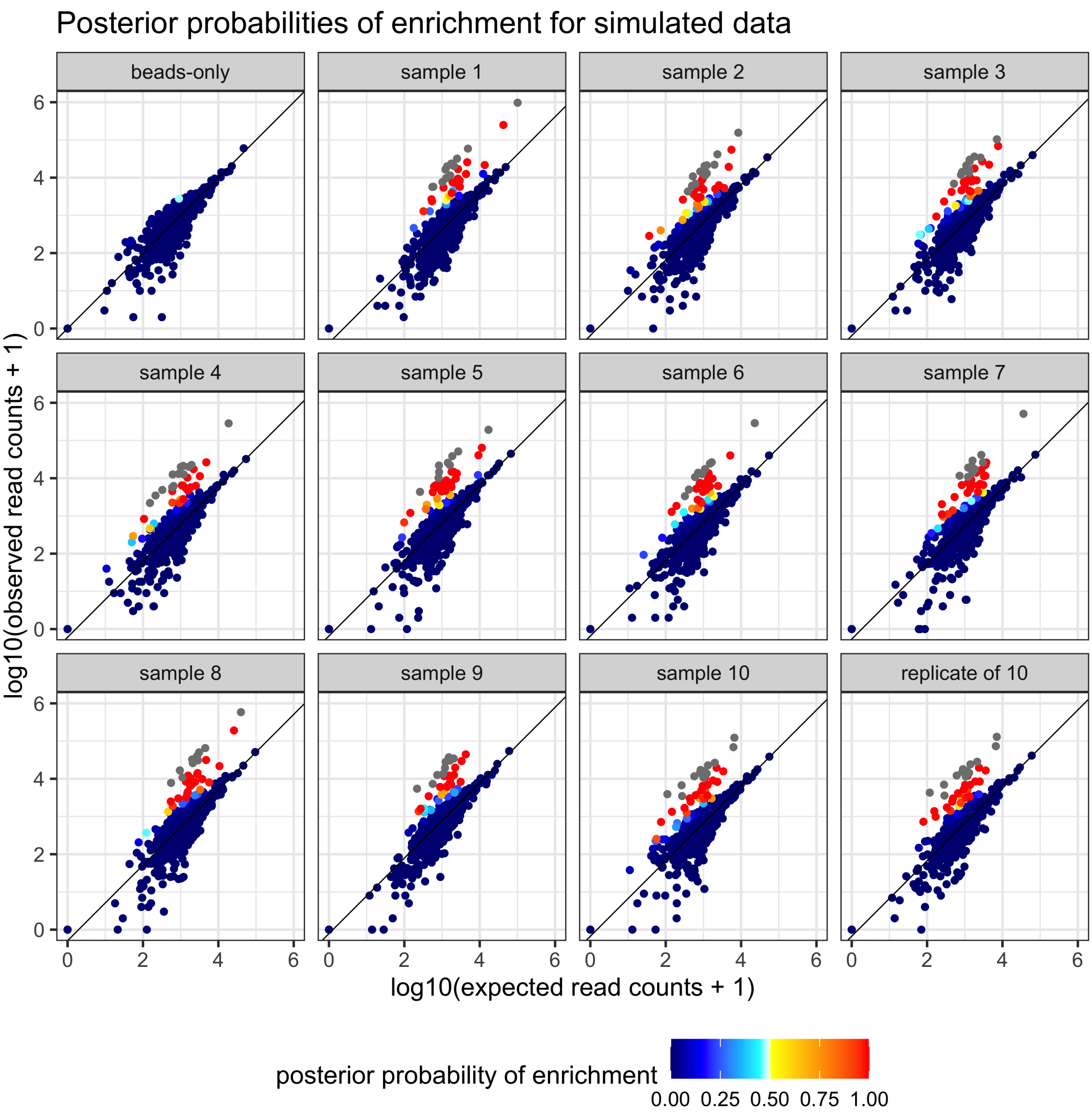

Figure S4: Posterior probability of enrichment for one simulated data set. Expected read counts for each peptide are derived by taking the average proportion of reads pulled in beads-only samples and multiplying the proportion by the library size of the sample. Peptides categorized as highly enriched are colored in grey. Warmer colors indicate that the peptide has over a 50% chance of being enriched. Points are plotted such that points with posterior probabilities closer to 0.5 are on top. The beads-only sample in the top left is run as a serum sample.

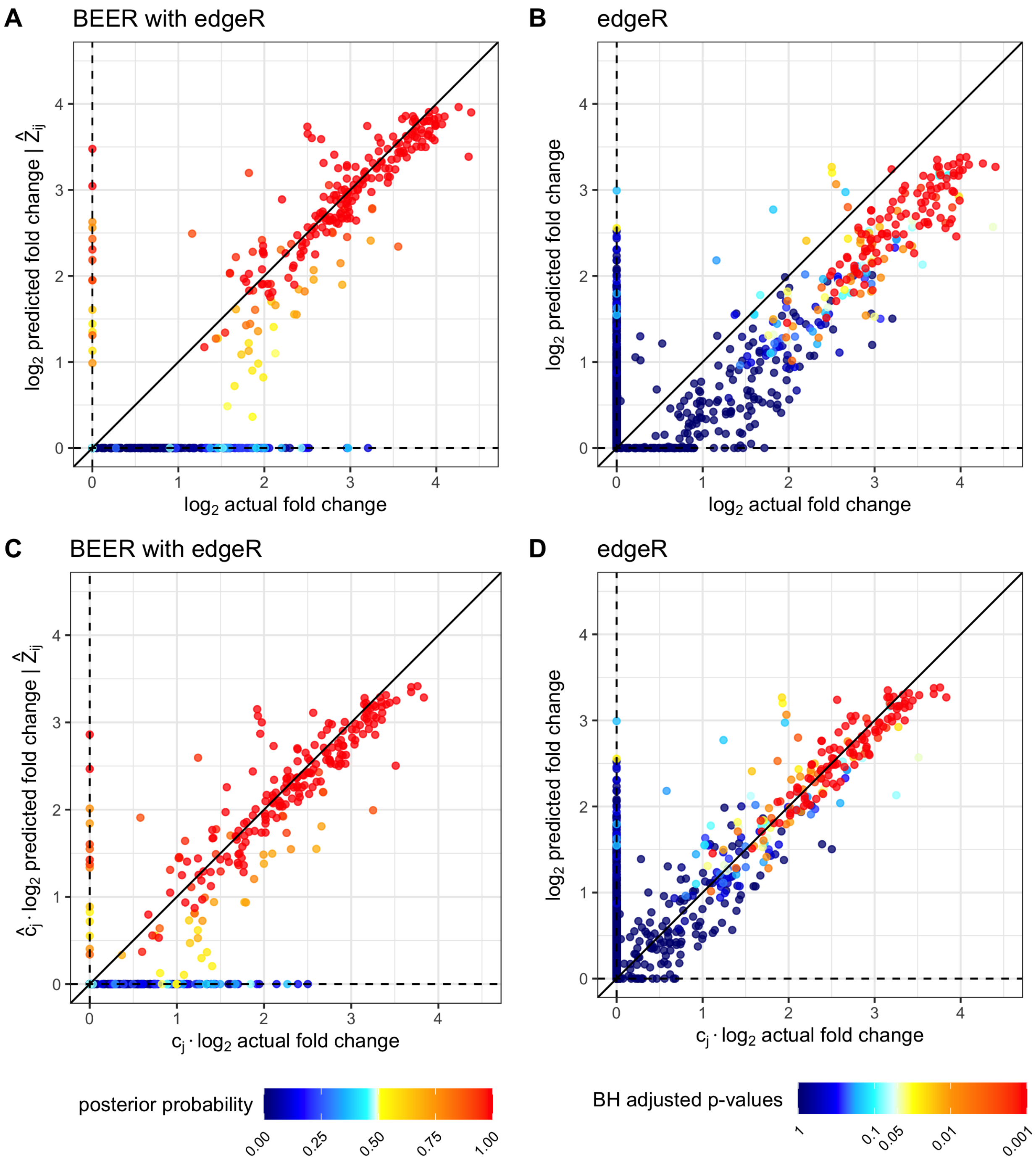

Figure S5: Comparison of estimated fold-changes to true fold-changes (A-B) and estimated fold-changes versus true fold-changes after adjusting for the attenuation constant (C-D) for one simulated data set. Only peptides from serum samples are included in each plot, and each peptide is represented by a point. Note that by construction, there are 120 peptides between each $\log_2$ increment, and highly enriched peptides are not included in the above plots. Warm colors indicate high probability of enrichment (posterior probability of enrichment > 0.5 or $-\log_{10}(\text{p-value}) > 20$).

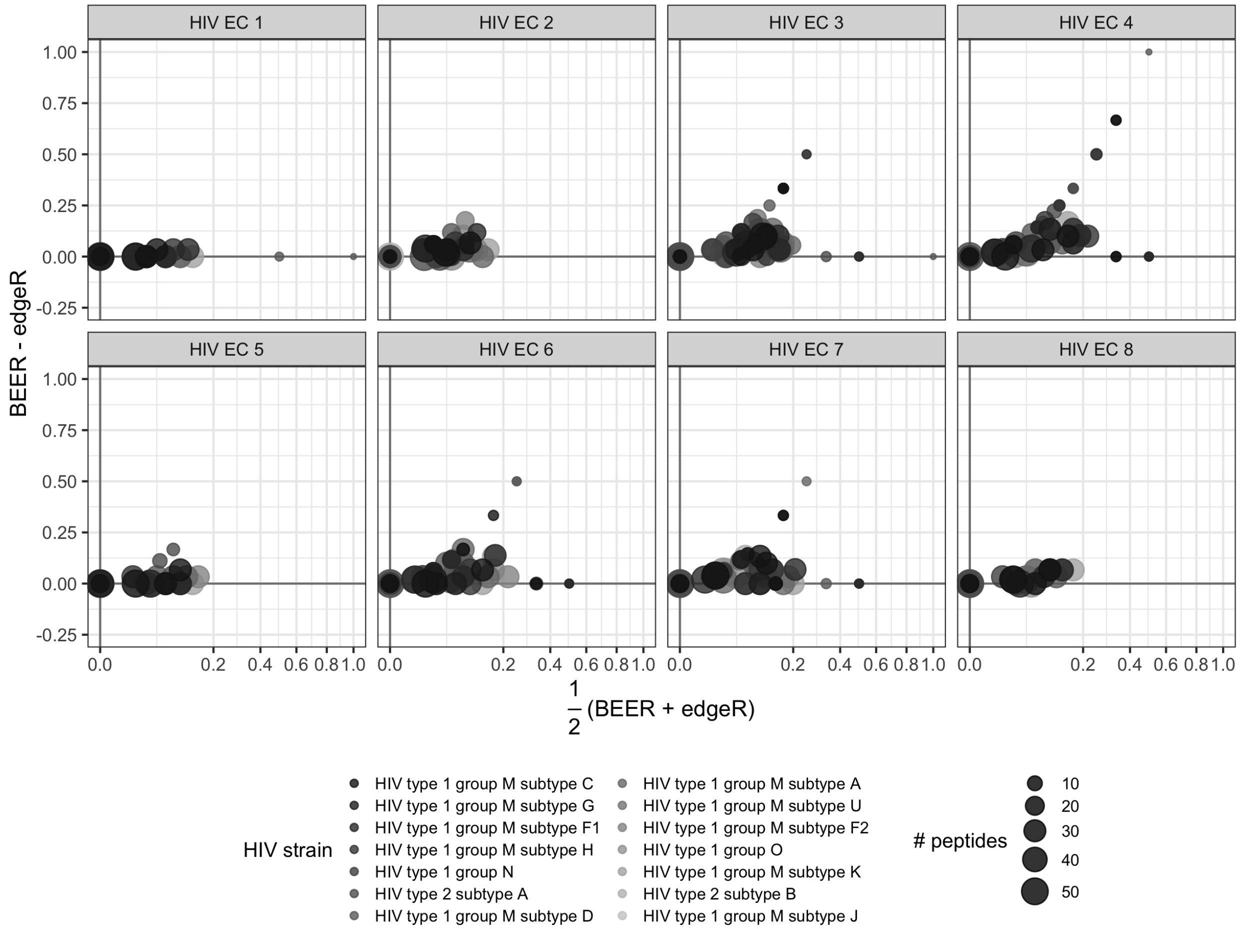

Figure S6: Proportion of enriched peptides by protein without HIV subtype B. Each point represents a protein. The color of the point indicates which virus the protein belongs to, and the size of the point corresponds to the number of peptides tiling the protein.

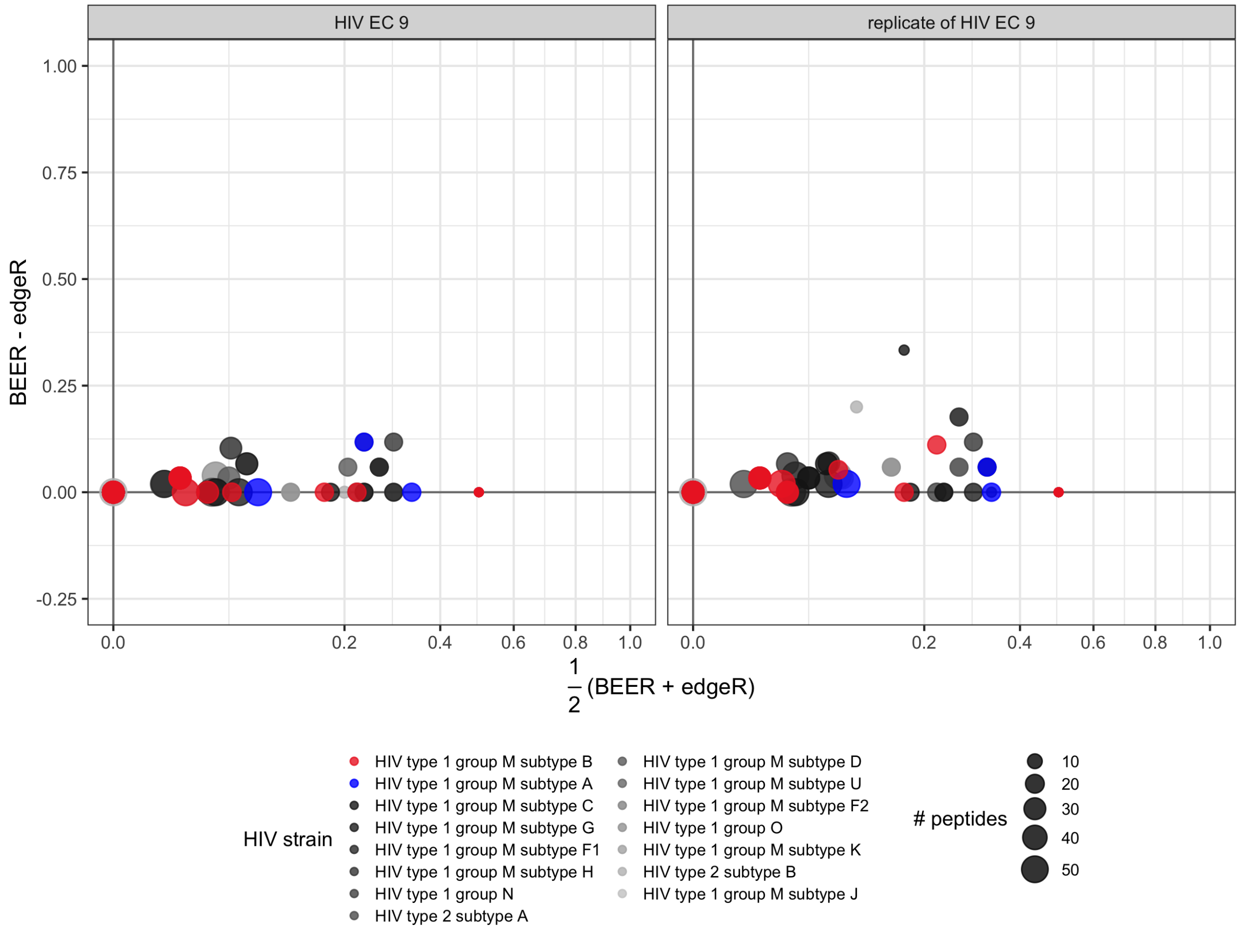

Figure S7: Proportion of enriched peptides by protein across technical replicates. This individual was infected with HIV subtype A. Each point represents a protein. The color of the point indicates which virus the protein belongs to, and the size of the point corresponds to the number of peptides tiling the protein.

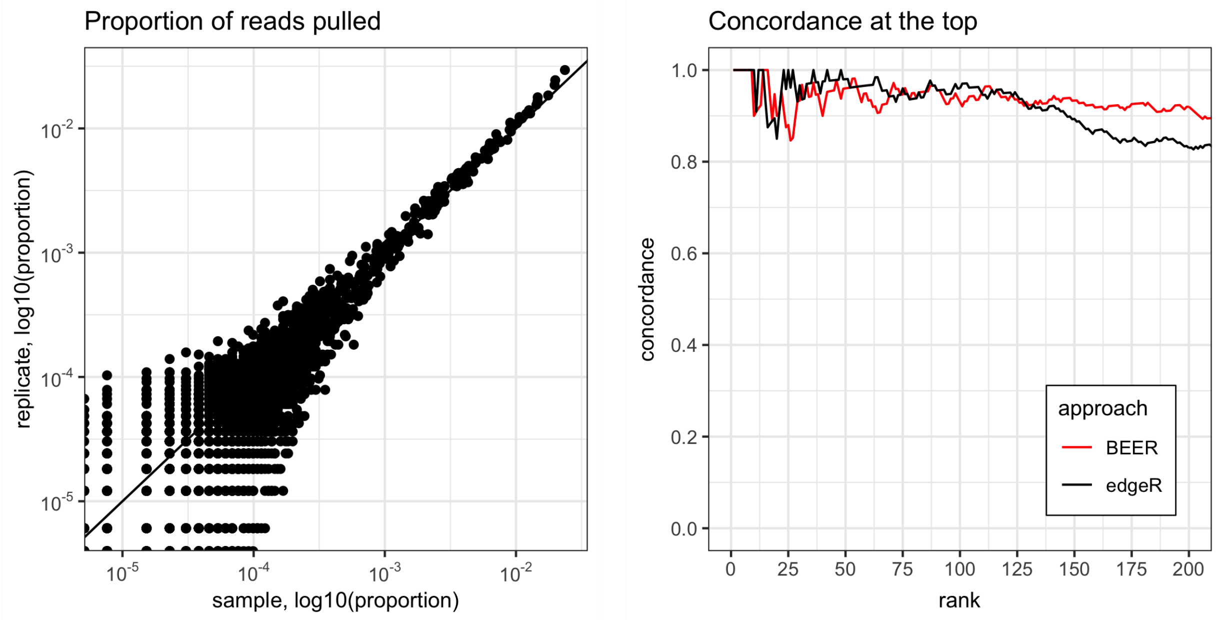

Figure S8: Left: proportion of reads pulled for 3,395 HIV peptides for two technical replicates. Right: concordance of HIV technical replicates, shown as proportion of peptides among the top ranked peptides in both replicates. For BEER, peptides are ranked by decreasing posterior probability of enrichment. For edgeR, peptides are ranked by increasing p-values. For both methods, ties of posterior probabilities and p-value (e.g., 0 and 1) were broken by the estimated fold-change. The top eight peptides from BEER are all highly enriched and treated exchangeably as no fold-change estimates are returned.

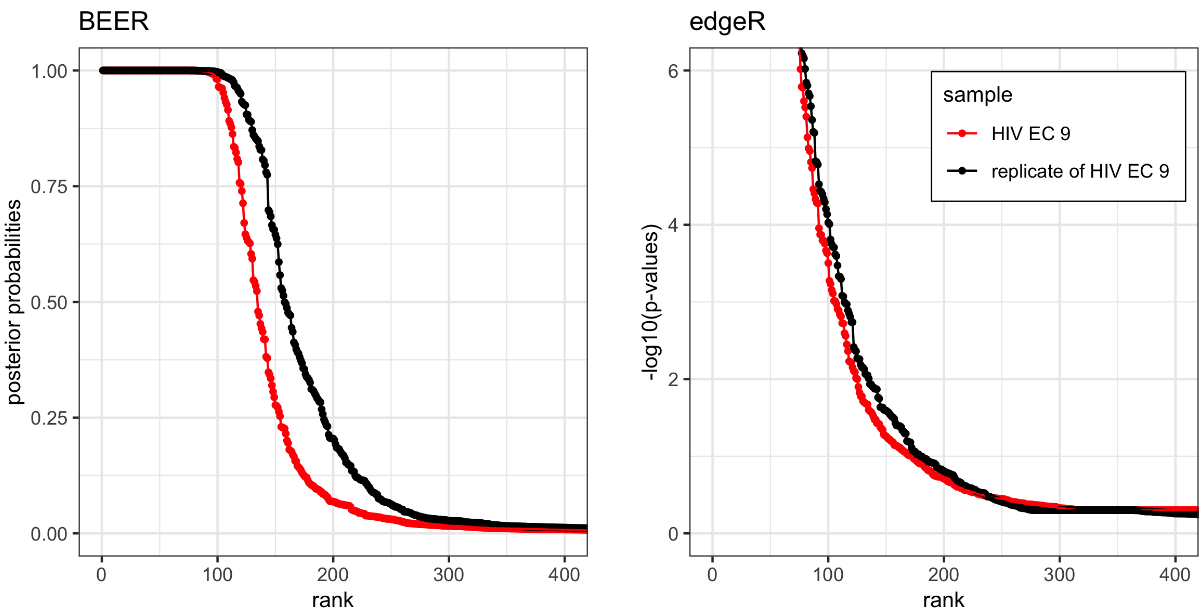

Figure S9: HIV replicate posterior probabilities by rank. For each of the technical replicates, peptides are sorted in decreasing order by posterior probability and -log10(edgeR p-values). For clarity of display, p-values were truncated at 10^{-6}.

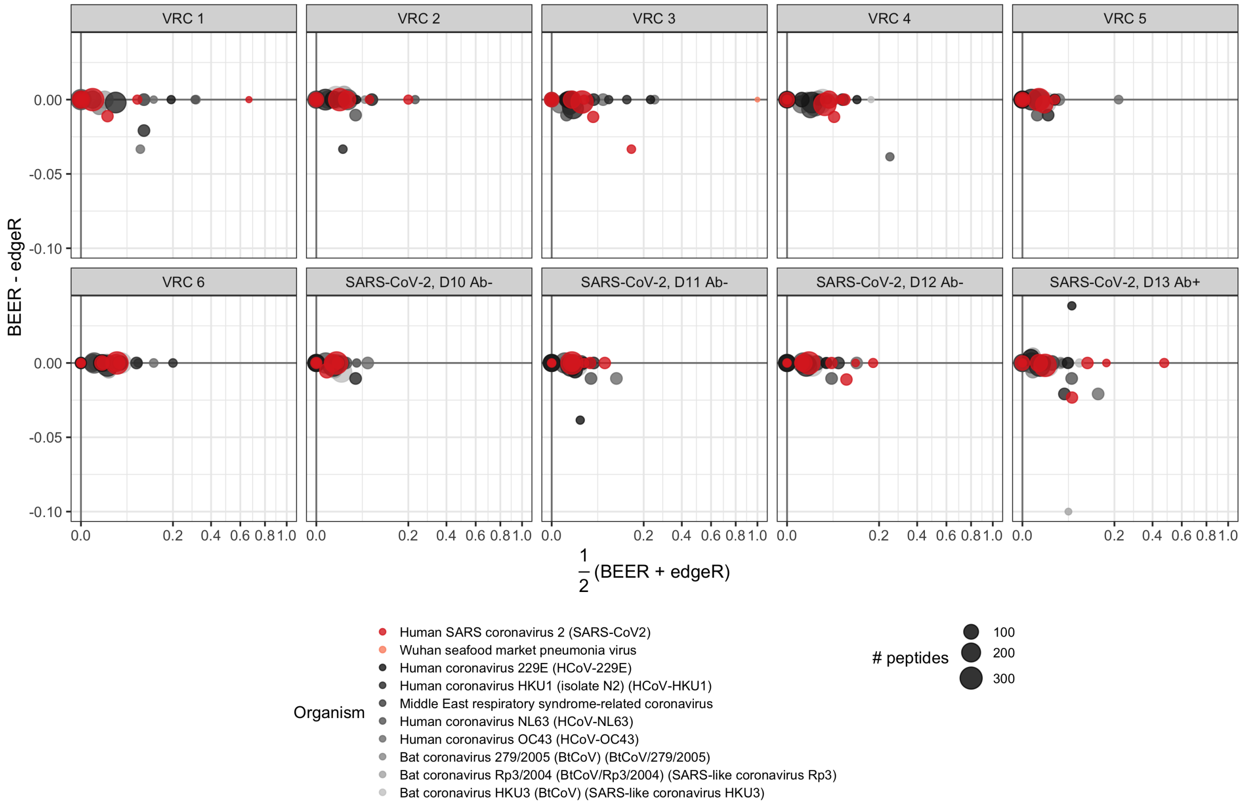

Figure S10: MA plots for the proportion of enriched peptides by protein for six pre-pandemic samples and four samples from one individual infected with SARS-CoV-2. Samples from this individual were collected at various days since symptom onset (labels D10-D13) and were additionally tested for SARS-CoV-2 antibodies. Antibody test results (positive or negative) are indicated by Ab+ or Ab-, respectively. Points represent individual proteins; point colors indicate virus types; and point diameters indicate the number of peptides tiling the respective proteins. In the CoronaScan library, peptides are present in duplicate, so the number of peptides is double the number of unique peptides.

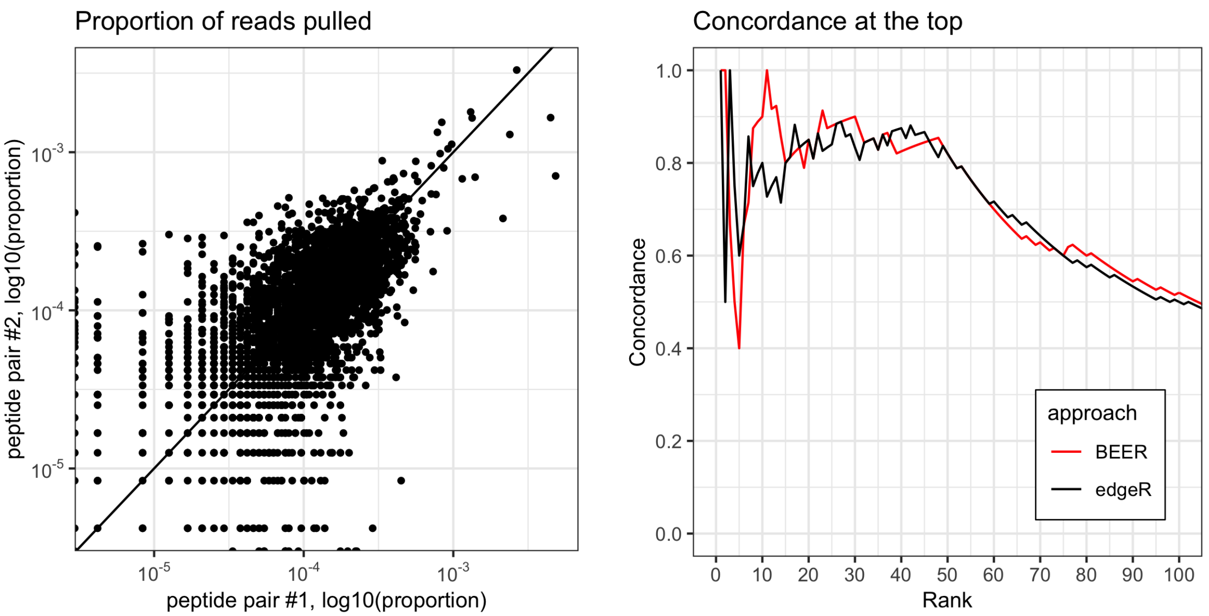

Figure S11: Left: concordance of paired peptides in CS sample VRC 1. For each unique peptide sequence, the proportion of reads pulled for peptide 1 with the same sequence is plotted against the proportion of reads pulled for peptide 2 of the same sequence. Right: concordance between the rankings for the top k ranks (x-axis) between all peptide pairs. For BEER, peptides are ranked by decreasing posterior probability of enrichment, with ties broken by the estimated fold-change (red line). For edgeR, peptides are ranked by increasing p-values, with ties again broken by estimated fold-changes (black line).

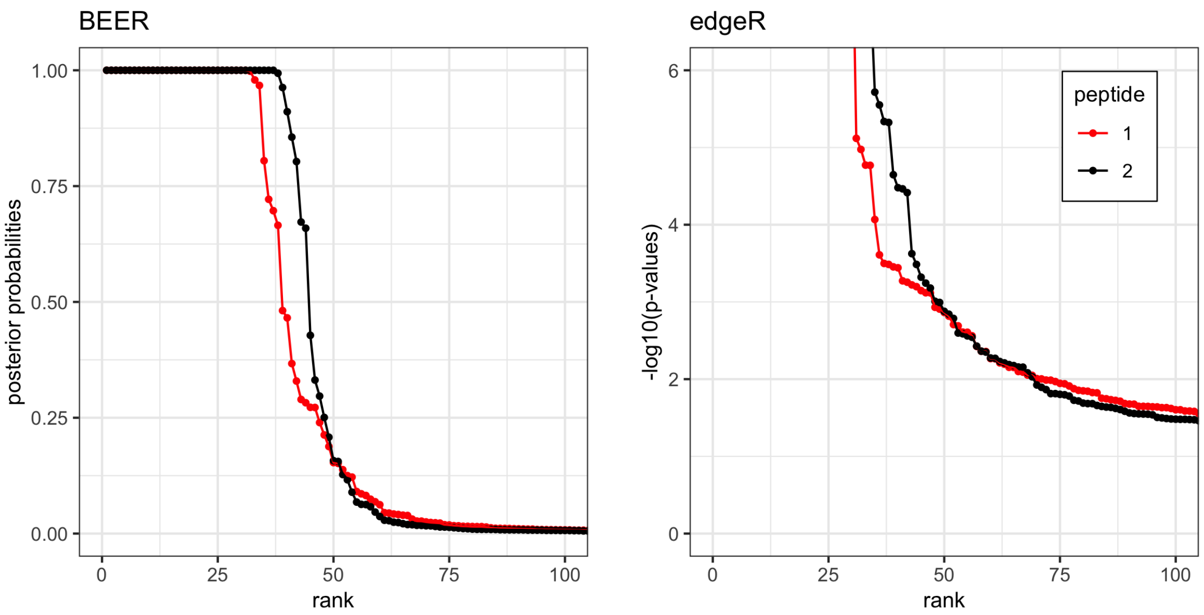

Figure S12: CoronaScan peptide pairs by rank. For each set of unique peptides, peptides are sorted in decreasing order by posterior probability and -log10(edgeR p-values). For clarity of display, p-values were truncated at 10^{-6}.

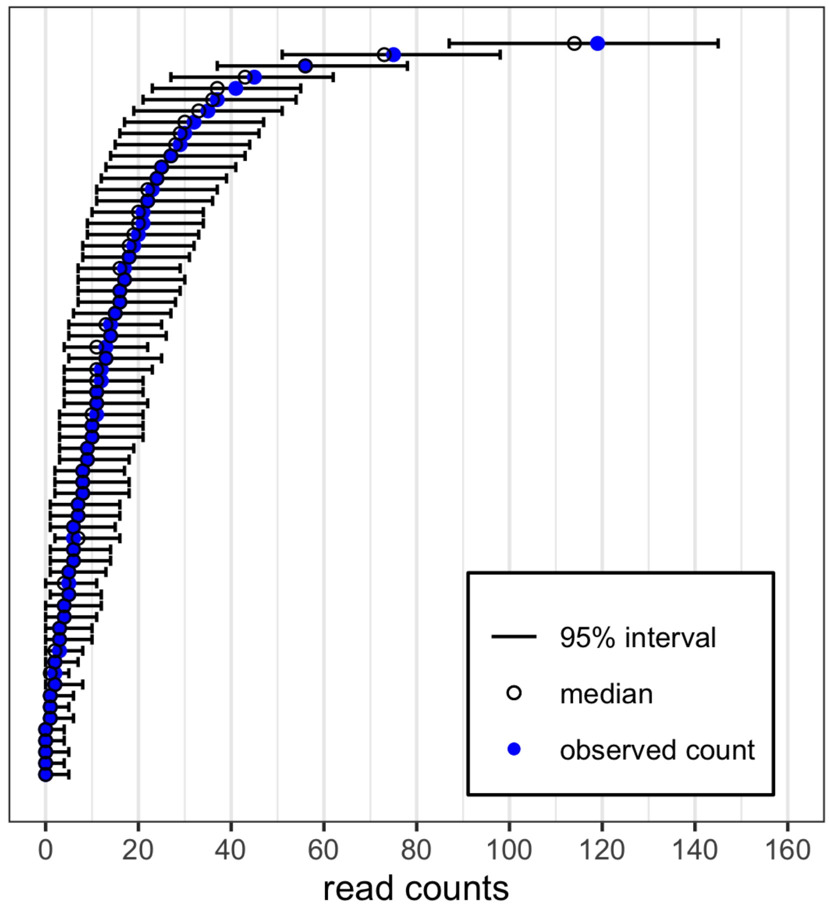

Figure S13: Posterior predictive 95% credible intervals for HIV EC 1 (horizontal lines) and medians (black circles) from the posterior predictive distribution for 67 of 3,394 HIV peptides, compared to the observed read counts (blue points). The peptides were chosen by ordering the observed read counts and selecting every 50th peptide.

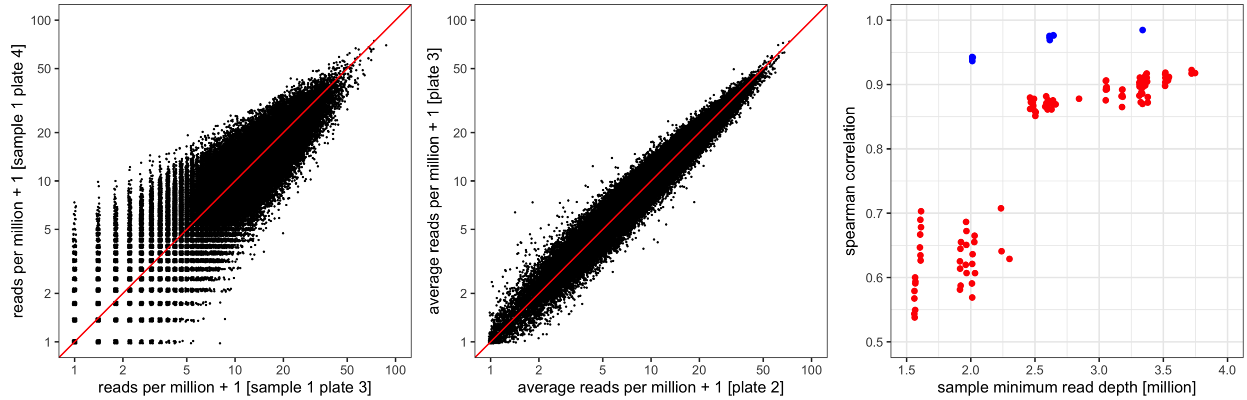

Figure S14: Evidence for a strong peptide effect in PhIP-Seq data, demonstrated using data from five plates of a previous experiment using HIV samples, analyzed in Eshleman et al.[18] and Chen et al.[35] Left: observed read counts per million reads for 95,242 peptides from two "beads only" samples from different plates. For these two samples, the Spearman correlation is 0.875. Middle: observed average read counts per million reads for 95,242 peptides from all "beads only" samples from two different plates. For these two plates, the Spearman correlation is 0.975. Right: within and between plate sample correlations as a function of sequencing depth. For each pair of bead only samples from the same plate (red dots, 117 pairs total), the Spearman correlation (y-axis) is related to the minimum of the respective two sequencing depths (x-axis). For each pair of plates (blue dots, 10 pairs total), the Spearman correlation between the average read counts of the bead only samples (y-axis) is also related to the minimum of the two median sequencing depths (x-axis), and substantially higher than the correlations of the bead only samples.

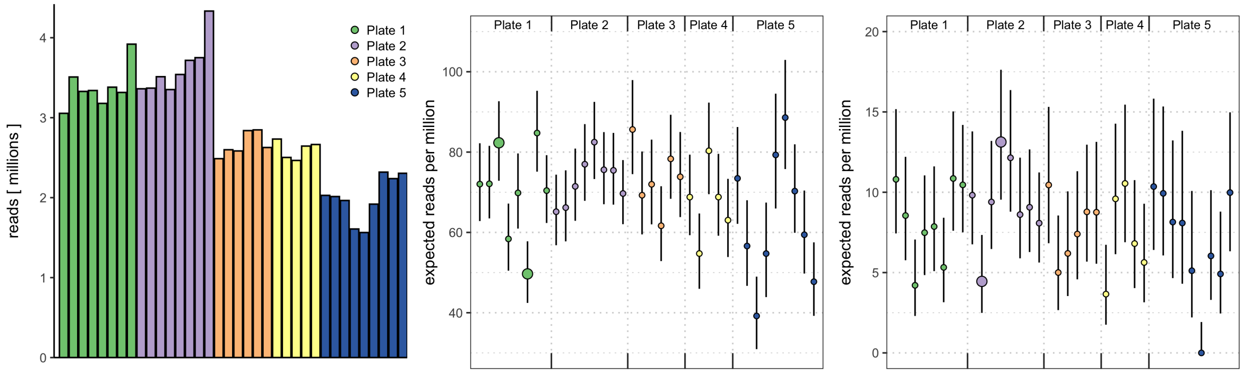

Figure S15: Evidence for larger than binomial variability in the PhIP-Seq data analyzed in Eshleman et al.[18] and Chen et al.[35] Left: library size (reads in millions) for 36 control ("bead only") samples from 5 plates. Middle: expected read counts per million reads aligned based on the estimated Binomial probabilities (dots, colored by plate) and respective 95% confidence intervals for a peptide with large expected read counts, for each of the control samples. Highlighted are samples 3 and 6 from plate 1, showing large discrepancies between the Binomial probabilities for this peptide between the two bead only samples. Right: the same statistics as in the middle panel, for a peptide with smaller expected read counts. Highlighted are samples 10 and 12 from plate 2, again showing a large discrepancy between the Binomial probabilities for this peptide between the two bead only samples.

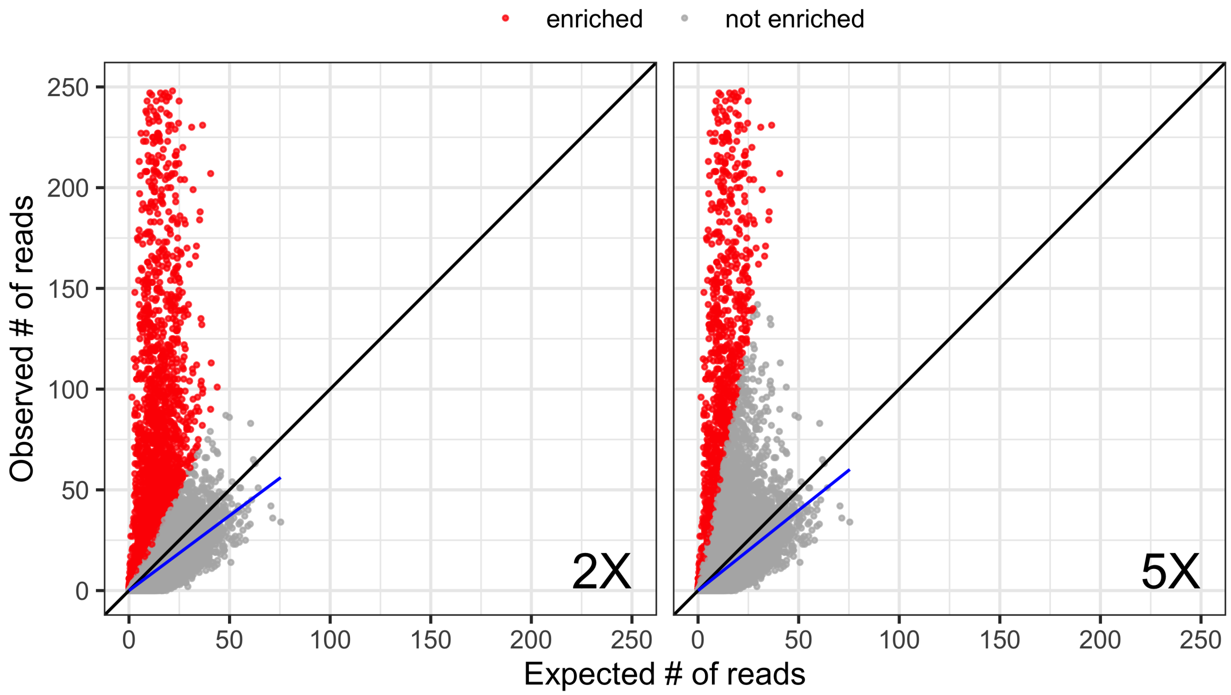

Figure S16: Expected versus observed read counts for 95,242 peptides from a randomly selected serum sample. Expected read counts for each peptide were derived using maximum likelihood estimates from the negative controls on the same plate. Each point represents one peptide from one sample, and peptides were considered enriched (red) if the observed read count was over 2 times (left) and 5 times (right) the expected number of reads. Linear regression lines (blue) were fitted using the non-enriched peptides and compared to the line where observed and expected reads are equal (black). The observed read counts were truncated at 250 to enhance the display.

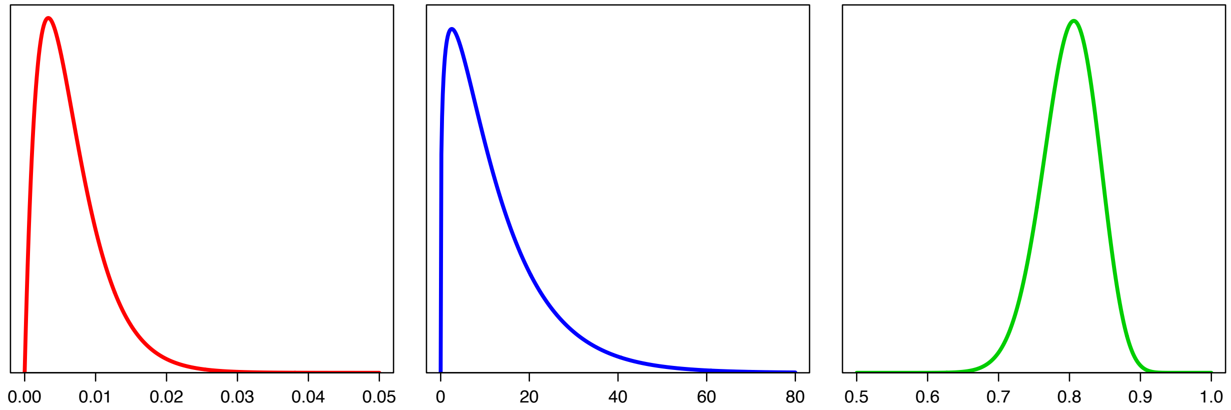

Figure S17: Left: the prior distribution for the proportion of reactive peptides in sample j, π_j, modeled as a Beta distribution Beta(a_π = 2, b_π = 300), reflecting peptide enrichment seen in previous studies. Middle: a Gamma(a_ϕ = 1.25, b_ϕ = 0.1) distribution, used in the prior distribution for the fold change ϕ_{ij} for peptide i in sample j, if reactive. Right: the prior distribution for the scaling constant in sample j, c_j, modeled as a Beta distribution Beta($a_c = 80$, $b_c = 20$).
